# ANOM Approach for Statistical Evaluation of Some Antioxidant Enzyme Activities

**DOI:** 10.3389/fchem.2022.894547

**Published:** 2022-05-26

**Authors:** Canan Demir, Sıddık Keskin, Fatih Şen

**Affiliations:** ^1^ Department of Biostatistics Zeve Campus, Faculty of Medicine, Van Yuzuncu Yil University, Van, Turkey; ^2^ Department of Biochemistry, Faculty of Arts and Science, Dumlupınar University, Kutahya, Turkey

**Keywords:** ANOM, free radicals, MDA, NLPCA, oxidative stress

## Abstract

Free radicals are chemical molecules that are more reactive and have an unpaired electron. Free radicals formed inside the cell oxidize biomolecules, leading to cell death and tissue damage. Antioxidants are molecules that can stabilize or inactivate free radicals before they damage the cell. In this study; the availability of Malondialdehyde, Superoxide dismutase, Catalase and Reduced glutathione levels as markers for related diseases was evaluated by examining whether and in what range they may vary in some diseases. In study, nine groups consist of prostate cancer, cirrhosis, liver transplantation, chronic kidney damage, acute kidney injury, X-ray exposure, CT exposure, MR exposure and Osteonecrosis. Analysis of means is a method developed to compare group means with the overall mean and presents the results graphically in an easy-to-understand manner without the required for any post hoc test. In addition, related characteristics were categorized as “low and high” and Nonlinear Principal Component Analysis was conducted to visually present their relationship with related disease types in two-dimensional space. The upper and lower decision lines were found 3.123 and 2.794 μmol/L, respectively for Malondialdehyde. Those with cirrhosis, chronic kidney disease, acute kidney disease and tomography exposure were included in the upper and lower decision lines. Those with prostate cancer, osteonecrosis, and X-ray exposure were above the upper decision line and are found higher than the overall mean. Those with lung transplantation and MR exposure appear to be below the lower decision line and lower than the overall mean. The present study provides the first comprehensive assessment of the availability of Malondialdehyde, Superoxide dismutase, Catalase and Reduced glutathione levels as markers for some related diseases. This study has shown that Analysis of means can be used as an alternative graphical procedure for multiple group comparisons with an overall mean in the studies regarding as biochemical characteristics and relating diseases. In addition, Nonlinear Principal Component Analysis can be useful aid for decision marker in some biochemical characteristics and related diseases.

## Introduction

Free radicals are chemical molecules that are more reactive and have an unpaired electron. It is constantly produced in cells as a by-product of metabolism. Free radicals formed inside the cell oxidize biomolecules, leading to cell death and tissue damage (Cheeseman et al., 1993). Free radicals work to oxidize the unsaturated fatty acids on the catalyzed membranes, and this process is called lipid peroxidation. Malondialdehyde (MDA) is a marker for oxidative stress and is one of the end products of lipid peroxidation. The malondialdehyde level reflects the degree of lipid peroxidation. An increase in free radicals causes overproduction of MDA (Gaweł et al., 2004; Koçak et al., 2020). Lipids, proteins, carbohydrates and other cell components are subject to oxidation, which is important in cell structures. The accumulation of this damage is called oxidative stress (Meydan et al., 2020). Oxidative stress occurs as a result of any imbalance between antioxidants and oxidants (Duzenli et al., 2019).

Free radicals (FR) are atoms containing an unshared electron pair. Free radicals are constantly produced in living cells. They contain both reactive oxygen species (ROS) and reactive nitrogen species (RNS). The importance of free radicals at physiological levels is that they play important roles by participating in protective reactions such as phagocytosis, detoxification and apoptosis (García-Gasca et al., 2009; Kocak et al., 2022). It is known that reactive oxygen radicals play an important role in autoimmune diseases such as rheumatoid arthritis, diabetes mellitus, atherosclerosis, obesity, cancer, cardiovascular and hypertension (Çolak et al., 2020; Çolak and Pap 2021). The concept of antioxidant describes molecules that can stabilize or inactivate free radicals before they damage the cell (Halliwell 2007). Some of the important antioxidant compounds are superoxide dismutase, catalase, and reduced glutathione. Superoxide dismutase (SOD) is present in all cells that use oxygen and is aerobic. It plays an important role in defense against the superoxide radical formed as a result of the reactions (Komuroglu et al., 2021). SOD enzyme accelerates the reaction four times in biological systems (Michiels et al., 1994). It is found in peroxisomes and is involved in the conversion of hydrogen peroxide to water and oxygen (Matés et al., 1999). Catalase (CAT) is a powerful antioxidant enzyme and is also used in the removal of toxic hydrogen peroxide (Aebi 1974). Reduced glutathione (GSH) removes harmful reactive oxygen radicals GSH has a strong antioxidant role GSH catalyzes the reduction of oxidized glutathione to glutathione GSH reduced glutathione, which is one of the most important antioxidant molecules of the indoor environment, has much physiological importance such as detoxification of xenobiotics, transport of amino acids, keeping sulfhydryl groups in proteins in a reduced state, and acting as a coenzyme in some enzymatic reactions [Beutler et al., 1963; Aydin et al., 2021).

It is important to determine the variables that can affect the events or processes in nature or to solve their effect mechanisms, in terms of directing these events in the desired way. In this framework, one of the methods used to determine whether the relevant factor or factors are effective on the continuous variable of interest is an analysis of variance (ANOVA). Analysis of variance (ANOVA) is a widely used method when assumptions are met, and post-hoc tests are then applied to determine significant different groups. ANOVA generally compare the means with each other in pairs. In some cases, it may be desirable to compare means with the overall mean or with the population mean. In this case, analysis of means (ANOM) may be preferred. ANOM is a method that was first introduced by Ott (1967) and developed to determine whether there is a difference between factor levels or groups and the overall mean, and presents the results graphically in an easily interpretable way. ANOM was also published in the early 1980s, using it in production and quality control (Kalanka et al., 2018).

In this context, in this study; the availability of MDA, SOD, CAT and GSH levels as markers for related diseases was evaluated by examining whether and in what range they may vary in some diseases. In addition, related characteristics were categorized as “low and high” and NLPCA was conducted to visually present their relationship with related disease types in two-dimensional space.

## Materials and Methods

This retrospective single-center study includes data from nine different studies conducted in accordance with the 1989 Declaration of Helsinki and approved by the Ethics Committee. Necessary permission was obtained from Van Yuzuncu Yıl University, Faculty of Science, Biochemistry Laboratory for the study. The data of the diseases whose MDA, SOD, CAT and GSH levels were examined between September 2021 and December 2021 were collected from the biochemistry laboratory and included in the study. The research only included patients over the age of 18. In study, nine groups consist of prostate cancer, cirrhosis, liver transplantation, chronic kidney damage, acute kidney injury, X-ray exposure, CT exposure, MR exposure and Osteonecrosis.

SOD activity by ‏Popov et al., CAT activity by Aeibi, GSH level by Beutler et al. and MDA level was determined by Gutteridge (Beutler et al., 1963; Aebi 1974; Gutteridge 1995; Popov et al., 2003).

### Statistical Analysis

ANOM is a method developed to compare group means with the overall mean and presents the results graphically in an easy-to-understand manner without the required for any post hoc test. The method requires two assumptions: normal distribution and homogeneity of variances. There are three lines in the ANOM graph. The overall mean is in the middle of the chart. Above the overall mean is the upper decision line, and below one is the lower decision line. Group means are indicated by dots on the graph. The fact that any group mean is outside the lower and upper decision line indicates that this mean is significantly different from the overall mean (Keskin et al., 2021). Decision limits can be calculated by Eqs 1, 2 for the equal sample size. However, Eqs 3, 4 are used if the sample sizes are unequal (Yigit 2019).

$$UDL=\bar{Y}+h\left(\alpha ;k,N-k\right)\sqrt{MSE}\sqrt{\frac{k-1}{N}}$$ (1)

$$LDL=\bar{Y}-h\left(\alpha ;k,N-k\right)\sqrt{MSE}\sqrt{\frac{k-1}{N}}$$ (2)

$$UDL=\bar{Y}+m\left(\alpha ;k,N-k\right)\sqrt{MSE}\sqrt{\frac{N-n_{j}}{Nn_{j}}}$$ (3)

$$LDL=\bar{Y}-m\left(\alpha ;k,N-k\right)\sqrt{MSE}\sqrt{\frac{N-n_{j}}{Nn_{j}}}$$ (4)

In the above equations, $\bar{Y}$ is the overall mean; h and m are the ANOM critical table value; *α* is the level of significance; k is the number of groups; N is the total number of observations; *n*_*j*_ is the sample size of the *j.* group; MSE shows the mean square error (Yigit 2019).

The principal component analysis is dimension reduction technique of multivariate data analyses methods (Sousa et al., 2007). Principal component analysis is a linear method and requires some assumptions such as normality of variables and linearity of the relationships between the variables. Nonlinear Principal Component Analysis (NLPCA) is used when these assumptions are not met. This method provides to examine the linear or non-linear relationships. The method is an explanatory dimension reduction method that provides numerical and visual results for datasets containing continuous, categorical or discrete variables (Demir, 2021).

In the study, MINITAB (v. 14) and SPSS (v. 20) for Windows programs were used for statistical calculations.

## Results

Descriptive statistics for MDA, SOD, CAT and GSH levels are given in Table 1. As seen in Table 1, the smallest mean value for MDA is 1.983, the largest mean value is 3.666, and the overall mean is 2.958 ± 0.704. It was determined that the smallest mean value for SOD was 2.356, the largest mean value was 4.608, and the overall mean was 3.239 ± 0.787. The smallest mean value for CAT is 0.0452, the largest mean value is 0.0908, and the overall mean is 0.0696 ± 0.034. For GSH, the smallest value was 0.0026, the highest value was 0.0075, and the general average was 0.0049 ± 0.0025.

**TABLE 1:** Descriptive statistics for MDA, SOD, CAT and GSH levels.

	N	MDA (µmol/L) Mean ± Std. Dev	SOD (U/L) Mean ± Std. Dev	CAT (U/L) Mean ± Std. Dev	GSH (mmol/g) Mean ± Std. Dev
Prostate cancer	25	3.647 ± 0.482	4.064 ± 0.460	0.0747 ± 0.0018	0.00261 ± 0.00166
Liver transplant	30	1.983 ± 0.159	3.054 ± 0.944	0.0723 ± 0.0187	0.00416 ± 0.00273
Cirrhosis	30	3.002 ± 0.557	4.608 ± 0.873	0.0908 ± 0.0088	0.00412 ± 0.00223
Chronic kidney injury	30	2.821 ± 0.439	3.046 ± 0.355	0.0766 ± 0.0058	0.00398 ± 0.00280
Acute kidney injury	31	2.833 ± 0.424	3.062 ± 0.364	0.0755 ± 0.0005	0.00426 ± 0.00168
X-ray exposure	50	3.666 ± 0.355	2.356 ± 0.603	0.0525 ± 0.0204	0.00539 ± 0.00241
CT exposure	53	2.926 ± 0.457	3.215 ± 0.212	0.0763 ± 0.0006	0.00416 ± 0.00214
MR exposure	52	2.262 ± 0.234	3.203 ± 0.281	0.0761 ± 0.0005	0.00747 ± 0.00216
Osteonecrosis	45	3.443 ± 0.711	3.295 ± 0.544	0.0452 ± 0.0816	0.00564 ± 0.00109
*p*-value		0.001	0.001	0.001	0.001
Total	346	2.958 ± 0.704	3.239 ± 0.787	0.0695 ± 0.0336	0.00491 ± 0.00251

**FIGURE 1:**
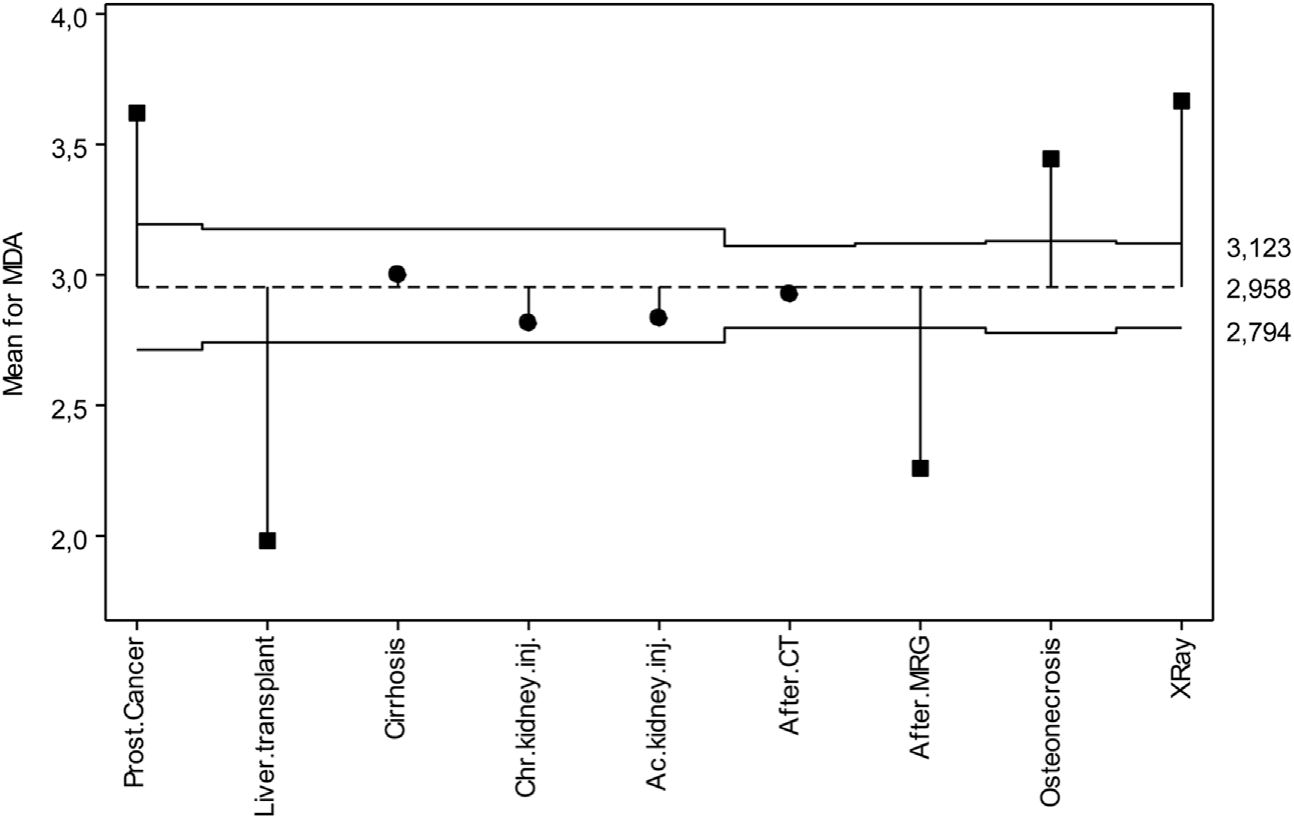
ANOM diagram for MDA levels.

On the other hand, the mean of each group was compared with the overall mean to determine statistically significant differences from the overall mean. As seen in Figure 1, the overall mean of MDA is 2.958 μmol/L, which is in the middle of the chart. The upper and lower decision lines were found 3.123 and 2.794 μmol/L, respectively. Those with cirrhosis, chronic kidney disease, acute kidney disease and tomography exposure were included in the upper and lower decision lines. Those with prostate cancer, osteonecrosis, and X-ray exposure were above the upper decision line and are found higher than the overall mean. Those with lung transplantation and MR exposure appear to be below the lower decision line and lower than the overall mean.

**FIGURE 2:**
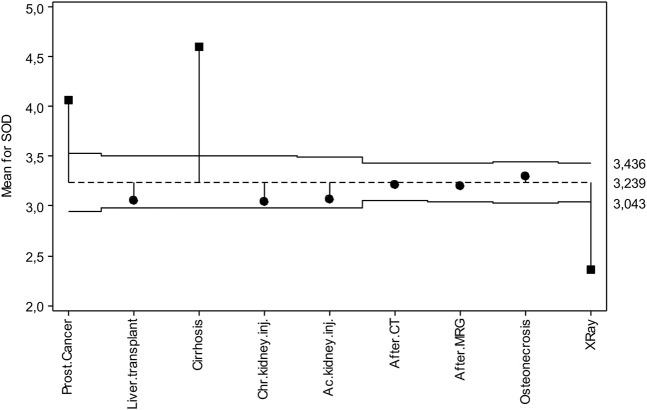
ANOM diagram for SOD levels.

Figure 2 shows the deviations from the overall mean for SOD. Accordingly, the overall mean of SOD is 3.239 U/L in the middle of the chart. Upper and lower decision lines are 3.436 and 3.043 U/L, respectively. In general, it is seen that six out of nine diseases are within the decision lines. These are lung transplantation, chronic kidney disease, acute kidney disease, tomography exposure, MR exposure and osteonecrosis. Prostate cancer and cirrhosis patients are in the upper decision line, and those with X-ray exposure are in the lower decision line.

**FIGURE 3:**
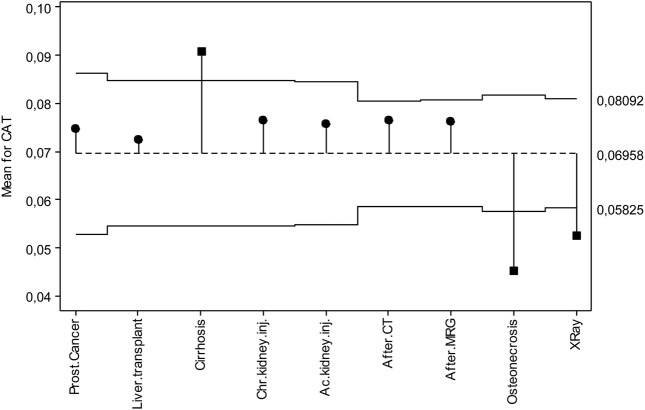
ANOM diagram for CAT levels.

Figure 3 shows the deviations from the overall mean for CAT. Accordingly, the CAT overall mean is in the middle of the chart with a value of 0.06958 U/L. The upper and lower decision lines are 0.08092 U/L and 0.05825 U/L, respectively. Those with prostate cancer, lung transplantation, chronic kidney disease, acute kidney disease, tomography exposure and MR exposure are among the decision lines, while those with cirrhosis are in the upper decision line, and those with osteonecrosis and X-ray exposure are in the lower decision line.

**FIGURE 4:**
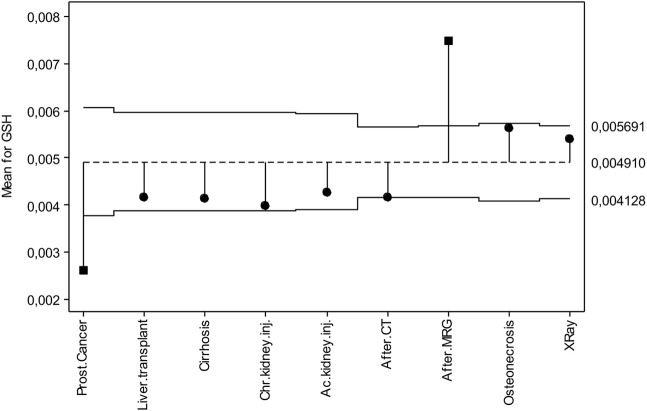
ANOM diagram for GSH levels.

Figure 4 shows the deviations from the overall mean for GSH. Accordingly, the general average of GSH is 0.00491 mmol/g. The upper and lower decision lines are 0.00569 mmol/g and 0.00412 mmol/g, respectively. While prostate cancer was in the lower decision line, those with MR exposure were in the upper decision line, those with lung transplantation, cirrhosis, chronic kidney disease, acute kidney disease, computed tomography exposure, Osteonecrosis and X-ray exposure took their place in the decision lines.

**FIGURE 5:**
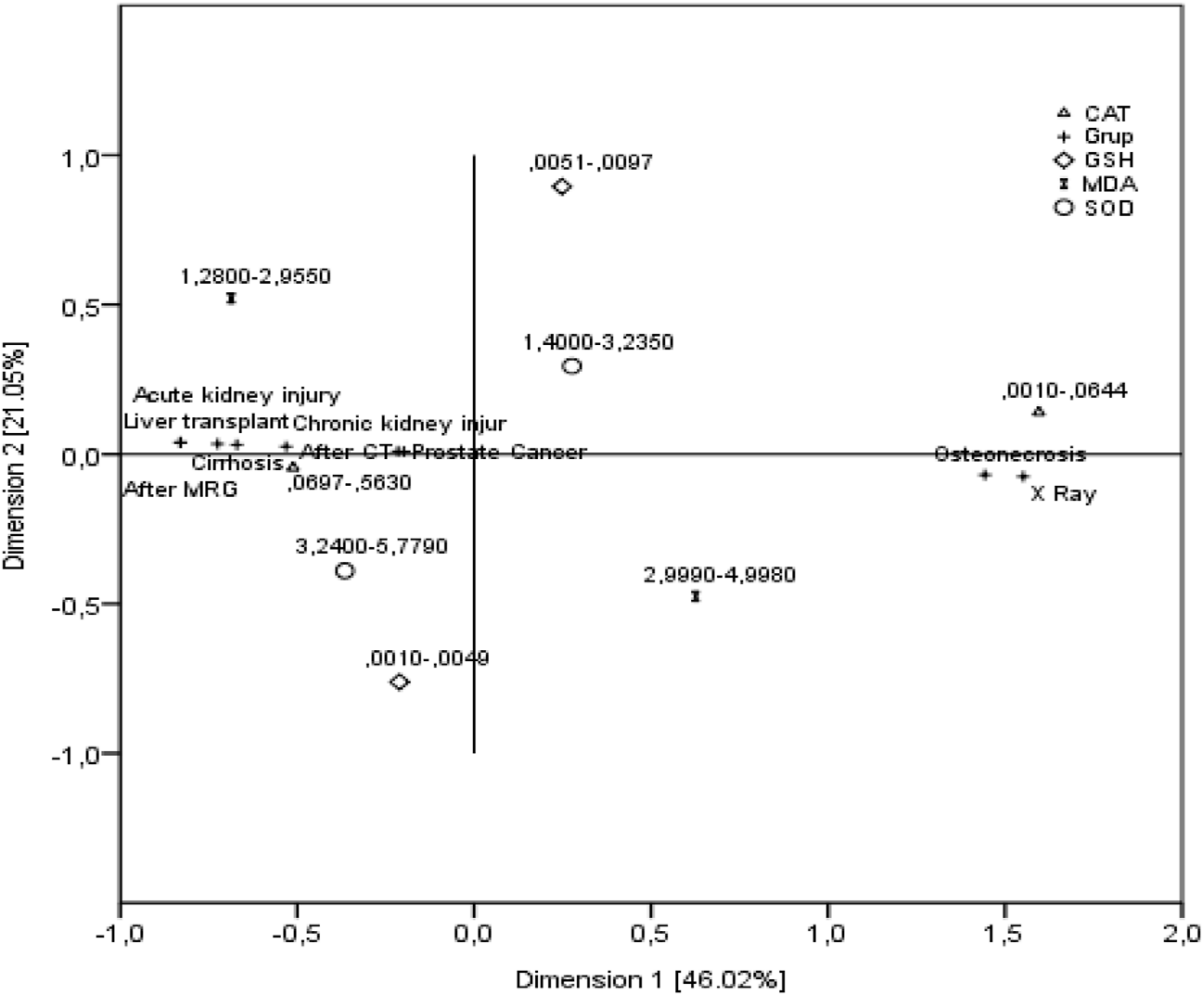
Configuration of groups and biochemical features in two-dimensional space.

The low and high categories of the considered features and the configuration of the groups in two-dimensional space are given in Figure 5. As seen in Figure 5, according to the NLPCA result, the first dimension accounted for 46% of the total variance, and the second dimension accounted for 21%. The two dimensions together explained 67% of the total variance.

As seen in Figure 5, in patients with osteonecrosis and X-ray exposure; high values of MDA level (2.999–4.9980) were associated with low levels of SOD (1.4-3.235), CAT (0.0010-0.0644) and GSH (0.0051-0.0097). Similarly, in patients with prostate cancer, lung transplantation, cirrhosis, chronic kidney disease, acute kidney disease, tomography exposure and MR exposure; low values of MDA (1.2800-2.9550) are associated with high values of SOD (3.2400-5.7790), CAT (0.0697-0.5630) and GSH (0.0010-0.0049).

## Discussion

In this study, MDA activity was below the lower decision line and SOD, CAT and GSH activity were between decision lines in liver transplant patients according to the ANOM test. In cirrhosis patients, MDA and GSH activities were between the decision lines, and SOD and CAT activities were above the upper decision line. In a study conducted in patients with liver transplantation, MDA, SOD and CAT activities were found above the upper decision line (Hassan et al., 2005). In another study conducted with male and female groups to determine the oxidative stress level in patients with liver disease, CAT and SOD activity were found above the upper decision line in both men and women (Monserrat-Mesquida et al., 2020). In a study conducted in cirrhosis patients, MDA activity was found below the lower decision line, while SOD, CAT and GSH activity were found above the upper decision line (Lai et al., 2020).

It has been observed that MDA and SOD levels are above the upper decision line, GSH level is below the lower decision line, and CAT level is between the decision lines in prostate cancer patients. In the study of Yilmaz et al. (2004), MDA level was found above the upper decision line in patients with prostate cancer (Yilmaz et al., 2004). In the study of Shukla et al., it was determined that the SOD value was below the lower decision line and the CAT and GSH value was above the upper decision line in patients with prostate cancer (Shukla et al., 2020).

ANOM visually presents whether the differences of the groups from the overall mean are statistically significant without any post hoc test. In ANOVA, multiple comparison testing is required to determine the difference between groups after the null hypothesis is rejected. However, ANOM can simultaneously identify these differences in a single step and present the results graphically. Ryan stated that ANOM can be used alone or after ANOVA (Ryan 2011). Nelson and Dudewicz emphasized that ANOM has some advantages in describing the differences from the overall mean and provides a graphical representation that helps evaluation thanks to its practicality (Nelson et al., 2002). Nelson et al. stated that if any of the groups are statistically different, ANOM shows exactly which ones are different (Nelson et al., 2005). In addition, ANOM provides a graphical representation that allows to easily evaluating both the statistical and practical significance of the differences. Pallman and Hothorn reported that ANOM and ANOVA can be applied to similar problems and therefore can be considered as alternatives to each other (Pallmann et al., 2015). Similarly, Balakrishnan emphasized that ANOM is conceptually very similar to ANOVA, however it presents the results visually in terms of decision lines and control charts, and therefore it can be more useful than ANOVA (Balakrishnan 2013). Kalanka et al. emphasized that the ANOM procedure is appropriate to use, however it is not as widely used as ANOVA because the mathematical basis of ANOM is more complex than that of ANOVA (Kalanka et al., 2018).

In this study, MDA, SOD, CAT and GSH activities were among the decision lines in patients with chronic and acute renal failure according to the ANOM test. In studies conducted with patients with chronic renal failure, MDA and SOD activity were found between decision lines (Vaziri et al., 2003; Ninić et al., 2018). In another study comparing the antioxidant activities of patients before and after dialysis, MDA activity was found between the decision lines, and SOD and CAT activities were found above the upper decision line (Tajbakhsh et al., 2017). In a study conducted with patients with acute renal failure, MDA activity was found above the upper decision line (Mishra et al., 2008).

In this study, according to the ANOM test; MDA activity was located between the decision lines in those with computed tomography exposure, below the lower decision line in those with MR exposure, and above the upper decision line in those with X-ray exposure. SOD and CAT activity were between the decisions lines in those with computed tomography and MR exposure, and below the lower decision line in those with X-ray exposure. GSH activity was at the lower decision line in those with computed tomography exposure, above the upper decision line in those with MR exposure, and between the decision lines in those with X-ray exposure. In a study conducted on people working in radiation environments, the effects of ionizing and non-ionizing radiation were found above the upper decision line of MDA level, which is an indicator of oxidative stress (Celik et al., 2016). After high-resolution computed tomography, CAT and GSH activity, which is a strong antioxidant enzyme in the blood of patients, was above the upper decision line (Bryll et al., 2019). In a study conducted on healthcare personnel exposed to ionizing radiation, MDA and SOD activities, which are indicators of oxidative stress, were found above the upper decision line (Bolbol et al., 2021).

In this study, according to the ANOM test; MDA activity was above the upper decision line, SOD and GSH activities were located on the decision lines, and CAT activity was below the lower decision line in Osteonecrosis patients. In a study in which oxidative stress levels were determined in osteonecrosis patients, MDA activity was found below the lower decision line and GSH activity above the upper decision line in both saliva and serum (Bagan et al., 2014).

## Conclusion

In this study, MDA, SOD, CAT, and GSH levels in some disease groups were evaluated using the ANOM and NLPCA methods. The mean of each disease group was compared with the overall mean to determine statistically significant differences. In addition, the relationships between diseases and categories (low and high) of the biochemical characteristics were analyzed by NLPCA, and the result was presented in two-dimensional space. The present study provides the first comprehensive assessment of the availability of MDA, SOD, CAT and GSH levels as markers for some related diseases. This study has shown that ANOM can be used as an alternative graphical procedure for multiple group comparisons with an overall mean in the studies regarding as biochemical characteristics and relating diseases. In addition, NLPCA can be useful aid for decision marker in some biochemical characteristics and related diseases. However further studies required to determine clearly the relations between MDA, SOD, CAT and GSH levels and these diseases.

## Data Availability

The original contributions presented in the study are included in the article/supplementary materials, further inquiries can be directed to the corresponding author.
